# A method for standardizing the fat content of human milk for use in the neonatal intensive care unit

**DOI:** 10.1186/1746-4358-4-3

**Published:** 2009-04-16

**Authors:** Charles Czank, Karen Simmer, Peter E Hartmann

**Affiliations:** 1 Discipline of Biochemistry and Molecular Biology, School of Biomedical, Biochemical and Chemical Science, The University of Western Australia, Crawley, Western Australia, Australia; 2 Neonatal Clinical Care Unit, King Edward Memorial Hospital, Subiaco, Western Australia, Australia

## Abstract

**Background:**

Accurately targeting the nutritional needs of the early preterm infant is challenging when human milk is used due to the natural variation in energy composition. The purpose of this study was to develop and evaluate a simple method for reducing the variation in fat and energy content of human milk prior to fortification such that the infant receives a diet of known composition.

**Methods:**

Milk was centrifuged at low speed to concentrate the fat into a cream layer and a predetermined volume of skim milk is removed to meet a specific fat concentration. The fat layer is then resuspended to produce reconstituted milk of a specified standard fat content.

**Results:**

Using this method it was possible to reduce the coefficient of variation in fat content of six different samples of donor human milk from 19.3% to 2.6%. As fat globule size may be associated with fat absorption, the effect that centrifugation and resuspension had on human milk fat globule distribution was assessed by laser diffraction particle sizing. No difference in the particle distribution of the treated and untreated human milk was observed.

**Conclusion:**

This method is accurate and simple, allowing for integration alongside current milk bank and NICU practices for use with both donor human milk and mother's own milk.

## Background

The benefits of using mother's own milk and donor human milk for premature and sick infants in the neonatal intensive care unit (NICU) are well known [1,2]. In particular, the use of human milk in the NICU is associated with decreasing the likelihood of infection and in turn reducing the length of stay in hospital and associated costs [3,4]. Unfortunately, human milk is not adequate to meet the nutritional needs of the early premature infant [5] and it is common practice to fortify the human milk prior to enteral feeding [6]. Fortification provides essential vitamins and minerals at necessary levels not ordinarily found in human milk, and is especially required to meet the protein and energy needs vital for adequate growth. Current recommendations of reasonable nutrient intakes state that protein:energy ratios of between 2.5–3.4 g protein/100 kcal of energy are required for extremely low birth weight (ELBW) infants and 2.6–3.8 g protein/100 kcal for very low birth weight (VLBW) infants [7]. However, given that the energy content of human milk varies widely [8], the desired protein:energy ratio may not be met, because the pre-fortification energy level of the human milk has not been standardized.

The total energy content of breast milk can be considered as the sum of the individual energy contributing components. Nutritionally, fat, lactose and protein are the most abundant energy sources contributing 9, 4 and 4 kcal/g (37.7, 16.7, 16,7 kJ/g) respectively [9]. Fat is the most variable component of human milk (40 ± 16 g/l [10], Coefficient of Variation (CV):40%) compared to lactose (63 ± 2 g/l[11], (CV):3.1%) and protein (9.2 ± 1.8 g/l [12], CV:19%) and varies between mothers, throughout the day and during a breast expression [8]. Often assumed human milk energy and protein content are used, which in turn may result in either a nutritional deficit once fortified or, conversely, a nutritional excess. The consequences of either under- or over- nutrition during this critical period of developmental programming may predispose the infant to a range of chronic disease states later in life [13-19].

As fat is the most variable nutritional component and contributes over half of the energy to breast milk [20], adjustment of the fat content to a specified level is a prerequisite to providing fortified human milk of a known energy content to meet the protein:energy needs of the preterm infant. The method described here allows for standardizing the energy content of human milk prior to fortification, such that all infants will receive a standard level of energy from breast milk.

## Methods

### Samples

Samples were obtained from a store of breast milk donated to the Perron Rotary Expressed Milk Bank (PREM Bank), Subiaco, Western Australia. Mothers had given prior consent for their milk to be used in research. All samples were collected by the mother and immediately frozen prior to transportation to the milk bank and research laboratory.

### Quantitation of fat content

The fat content of milk samples was determined by a spectrophotometric esterified fatty acid (EFA) method [8,21], as well as by using the creamotocrit method [22].

### Centrifugation

Samples of breast milk were thawed and 30 to 50 ml portions aliquoted into vessels. Skim milk and cream were separated by centrifugation at either 4°C or 10°C at a relative centrifugal force (RCF) and time needed to give a range between 125 to 12500 *g*.min (eg: 125 × *g *for 1 minute to 2500 × *g *for 5 minutes, respectively).

### Skim milk volume adjustment and resuspension of breast milk

**Figure 1:**
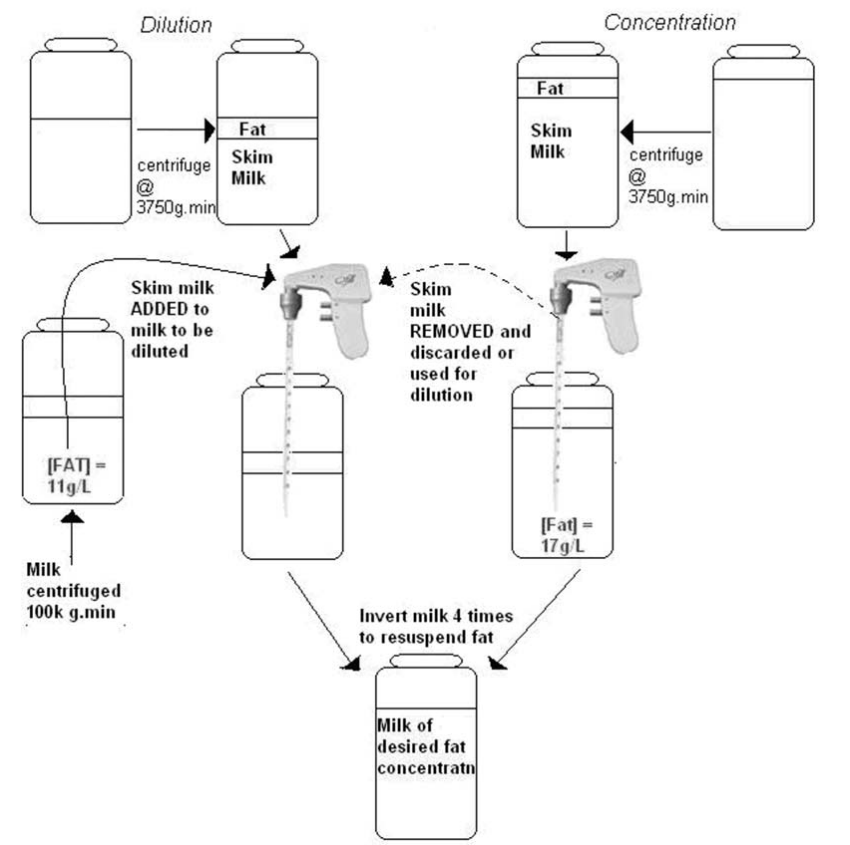
**Schematic representation of method used to standardize energy content of breast milk for use in the NICU**. Milk was firstly centrifuged at 3750 *g*.min at 4°C to concentrate the fat layer. A predetermined volume of skim milk was then removed if the fat concentration needed to be increased or added if the fat concentration needed to be decreased. The fat layer was then resuspended by four inversions of the vessel to produce reconstituted milk of specified fat concentration.

The volume of skim milk to be either removed or added was determined using the equation described below in the Results section. The container was then placed on a weighing scale and tared prior to adjustment and the required volume of skim milk adjusted carefully with a sterile pipette from below the fat layer (Figure 1). For those samples where skim milk was added to the milk, the skim was added above the cream layer prior to resuspension. The cream layer was then resuspended by inverting the container four times. All manipulations were performed at room temperature.

### Milk fat globule size distribution

Freshly expressed breast milk from a term mother was aliquoted into sterile 5 ml containers and either frozen at -20°C or stored at 4°C overnight. Frozen milk was then thawed and both the thawed and milk stored at 4°C mixed and 1.0 ml samples of mixed milk taken prior to centrifugation. Milk was then centrifuged at the 3750 *g*.min at 4°C and the cream layer resuspended and another 1.0 ml sample taken from the reconstituted milk. Particle size was determined using a Mastersizer 2000 fitted with Hydro SM sample dispersion system (Malvern Instruments). Absorbance was adjusted to meet a target weighted residual of 1%, a dispersant refractive index of 1.33 was used and sample added to the dispersion unit until an obscuration target of 10–15% was achieved. Averaged data from ten repeated scans was analyzed with Dispersion Technology Software V4.02 (Malvern Instruments).

### Statistical Analysis

All values were calculated in Microsoft Excel 2003 and expressed as mean ± standard deviation unless otherwise stated.

## Results

### Optimal relative centrifugal force for readily resuspending the cream layer

**Figure 2:**
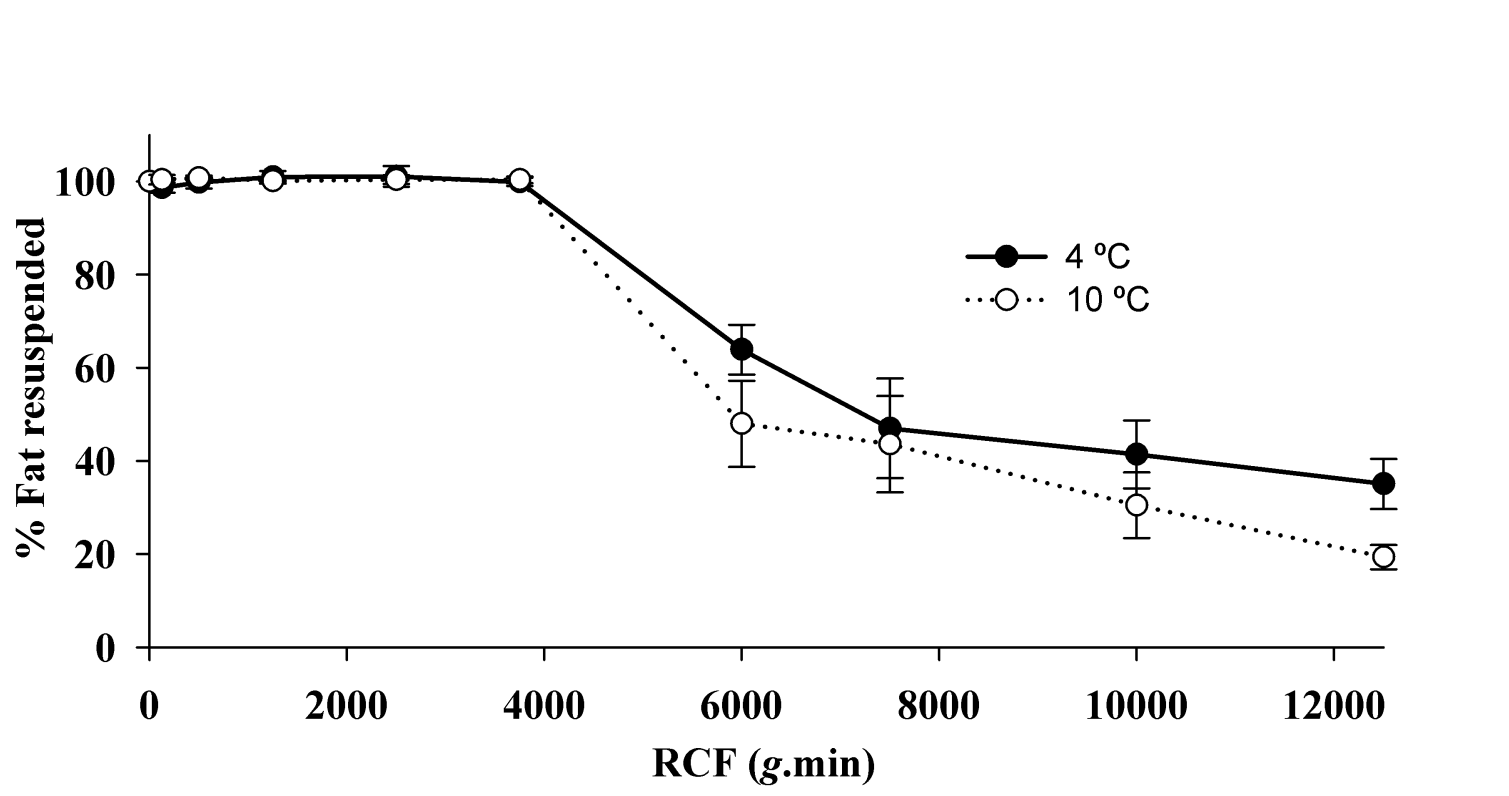
**Relationship between percentage of fat resuspensed by inversion of the container four times and relative centrifugal force (RCF)**. The maximum and therefore optimum RCF that allows for 100% fat resuspension is 3750 *g*.min. All data points are mean ± SD (N = 6)

**Figure 3:**
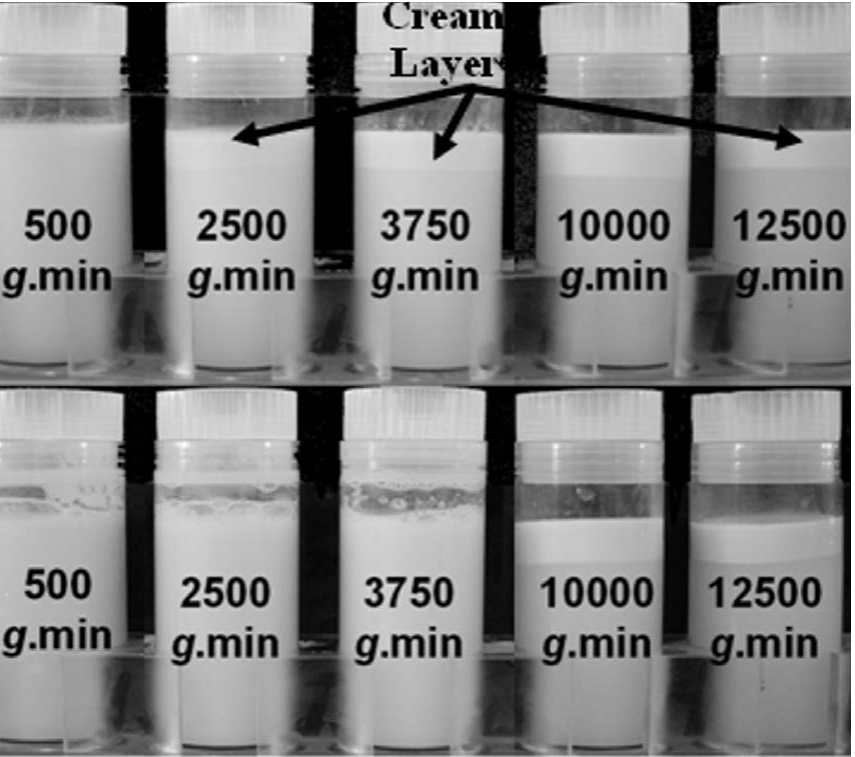
**Examples of milk centrifuged at different relative centrifugal forces ranging from 125 – 12500 *g*.min before (top row) and after mixing (bottom row) illustrating resuspension of the cream layer (labelled)**.

To allow the volume of skim milk to be adjusted to meet the specified total fat content, human milk was centrifuged to collect the cream in a layer at the top of the milk. It was necessary to determine the optimal RCF that allowed 100% fat resuspension, whilst still allowing the skim milk to be removed. This was achieved by centrifuging at RCF's ranging between 0 and 12500 *g*.min, sampling the skim milk, then resuspending the cream layer by four inversions of the centrifuged milk, followed by sampling of the reconstituted milk. By quantifying the fat content of the skim milk after centrifugation and of the reconstituted milk of six samples, it was determined that 3750 *g*.min was optimal for achieving 100% resuspension (Figure 2). When centrifuged at RCF's greater than 3750 *g*.min, either less than 100% fat resuspension was achieved or the fat layer did not resuspend (Figure 3). The effect of temperature on the resuspension of the fat layer was assessed by centrifuging at either 4°C or 10°C. However, no effect on the level of fat resuspension was observed (Figure 2).

### Fat content of skim milk after centrifugation

**Figure 4:**
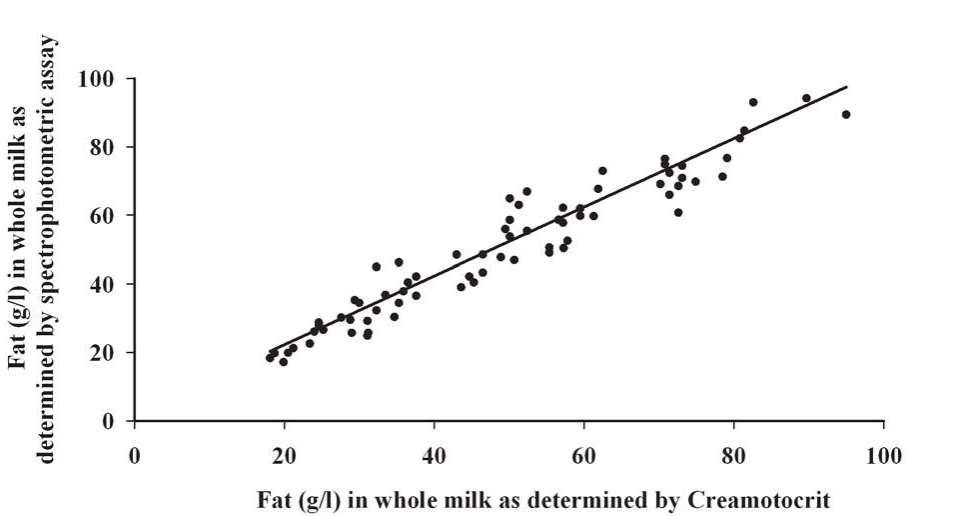
**Correlation between fat content of milk as determined using the creamotocrit and spectrophotometric methods**. (R2 = 0.93, n = 72).

**Figure 5:**
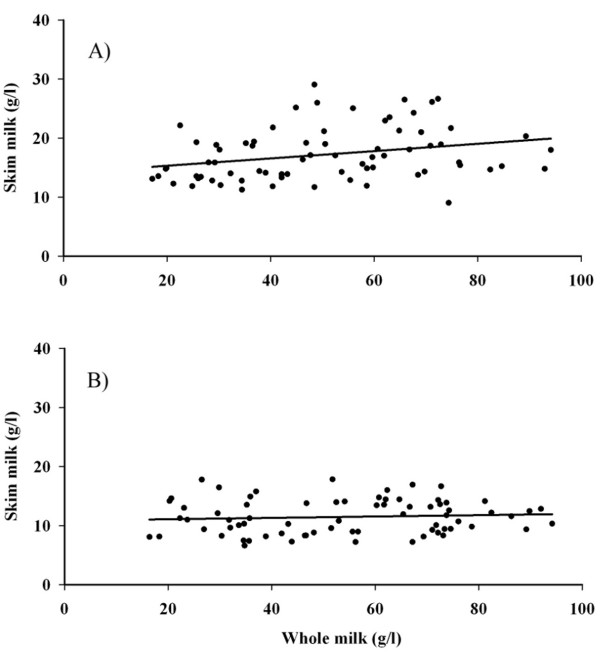
**Relationship between fat concentration of whole milk and skim milk centrifuged at optimum resuspension RCF (A: 3750 *g*.min) and at "defatting" (B: 100,000 *g*.min)**. The skim milk fat content averaged 17.2 ± 4.5 g/l (R^2 ^= 0.0772, n = 72) and 11.5 ± 2.9 g/l (R^2 ^= 0.0069, n = 72) for samples centrifuged at 3750 *g*.min and 100,000 *g*.min, respectively.

The fat content of the whole milk from six donors was determined by spectrophotometric assay and by creamotocrit with a mean ± SD of 49.2 ± 19.7 g/l (n = 72) and 53.92 ± 20.9 g/l (n = 72) respectively. A strong correlation (R^2 ^= 0.93) existed between the values determined by the two different methods (Figure 4). The fat content of the skim milk after centrifugation at 3750 *g*.min was 17.2 ± 4.5 g/l (n = 72). In comparison, the mean fat content of "defatted skim milk", produced by centrifuging for 100,000 *g*.min, was 11.5 ± 2.9 g/l (n = 72). The fat content of the skim milk did not correlate with whole milk fat content and remained relatively constant between samples (Figure 5).

### Development of an equation for skim milk volume adjustment to standardize breast milk fat content

The relationship between total fat content in the whole milk, fat content in the skim milk after centrifugation at 3750 *g*.min and final fat content after volume adjustment was used to develop an equation for skim milk volume adjustment to meet a specified fat content. The variables for this equation are defined below.

*V*_1 _= Initial volume of milk (ml)

*V*_2 _= Volume of skim milk to be adjusted (ml)

*V*_3 _= Final volume of milk after adjustment (ml)

*C*_1 _= Initial fat content (g/l)

*C*_2 _= Content of fat in skim milk to be added or removed (g/l)

*C*_3 _= Desired fat content (g/l)

Total grams of fat (*F*_*T*_) is defined as a function of volume and content

ie: 

Similarly, the total grams of fat in the skim milk (*F*_*S*_) is defined as: 

The total grams of fat in the final adjusted volume (*F*_*F*_) is defined as: 

As the final volume of milk and fat content was unknown, *F*_*F *_was expressed as a function of *F*_*T *_and *F*_*S *_as the difference between the total grams of fat in the initial volume and the total grams of fat in the skim milk that is added or removed:



*F*_*F *_can be expressed as a function of the difference of in the initial and skim volumes and the final fat content.

ie: 



Rearranged to give the skim milk volume to be adjusted (*V*_2_) gives:



### Proof of concept

Samples of milk from six mothers were used to validate this method and demonstrate that the natural variation between samples could be greatly reduced. The aim was to standardize the fat content of the samples to 49.3 g/l with no greater than 5% variation between samples. This value was equivalent to an energy content of 75 kcal/100 ml assuming protein content of 10 g/l and lactose of 67 g/l. The initial fat content of the individual mothers' milk are illustrated in Table 1 and ranged from 39.6 – 63.5 g/l (mean = 48.3 g/l, CV = 19.3%). The individual fat contents of the six different milk samples, the target fat content and the assumed skim milk fat content of 17 g/l were inputted into the equation described above to determine the amount of skim milk to be removed from or added to each sample (Table 1). After centrifugation at 3750 *g*.min for 4°C, the skim milk volume was adjusted and the fat layer resuspended for each sample. The cream layer was noticeably easier to pipette through if the samples were kept on ice when being manipulated. Conversely, the cream layer was much easier to resuspend once the samples had returned to room temperature. The fat content of the reconstituted milk fat ranged from 49.1 – 52.2 g/l (mean = 50.1 g/l, CV= 2.6%).

**Table 1:** Initial fat content, volume of skim milk adjusted and final fat content of milk from six mothers to which the fat standardization applied

Human milk sample	Initial fat content (g/l)	Volume of skim milk adjusted (ml)^a^	Final fat content of reconstituted milk (g/l)	Percentage difference from target fat content
1	48.8	-0.4	49.8	0.53%
2	39.6	-9.0	52.2	2.9%
3	40.3	-8.4	50.4	1.12%
4	63.5	+13.3	49.1	0.18%
5	43.3	-5.6	50.5	1.28%
6	54.6	+5.0	49.5	0.77%
Mean	48.3		50.1	
SD	9.4		1.3	
%CV	19.3		2.6	

a: as determined using the equation described in text: Plus (+) refers to skim milk added and minus (-) refers to skim milk removed, b: target specified fat content was 49.3 g/L, CV%: Coefficient of variation, SD: standard deviation. All samples were 30 mL in volume.

### Breast milk fat globule distribution in reconstituted breast milk after centrifugation and fat resuspension

**Figure 6:**
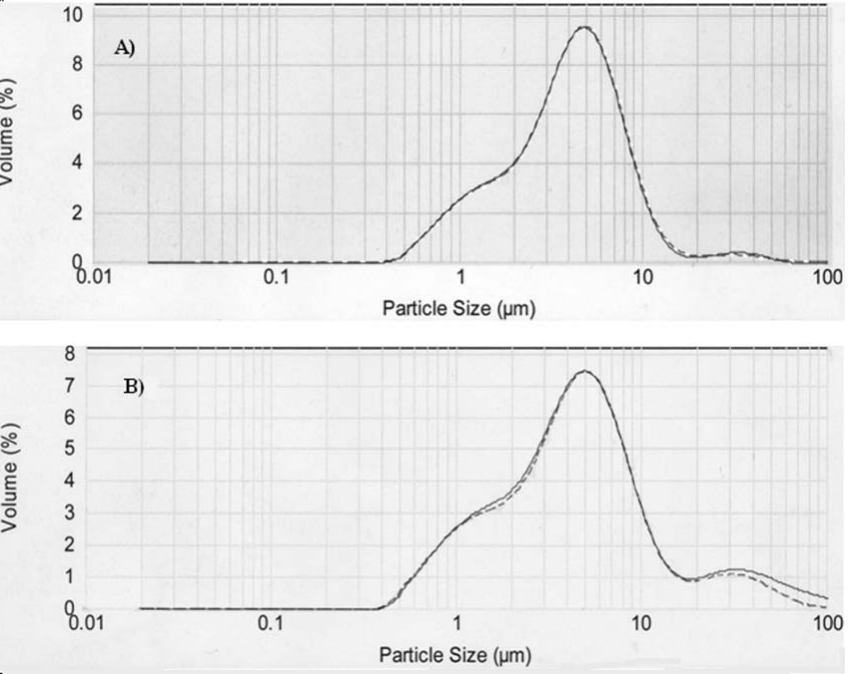
**Particle distribution of untreated (solid line) and reconstituted (dashed line) fresh (A) and frozen (B) human milk**.

Mean fat globule size ± SD was 3.25 ± 0.41 μm (n = 27). The fat globule distribution had a minor peak at 2 μm corresponding to a large number of small fat globules, a major peak at approximately 4 μm, representing the majority of fat globules of average size and shouldering off down to a minor peak at around 12 μm, representing a small number of very large globules. Frozen milk had a similar globule distribution to that of fresh milk except for slightly greater number of fat globules around 12 μm. Fat globule distribution was not affected in the reconstituted milk and very closely resembled the particle distribution of the untreated whole milk for both the fresh and frozen samples (Figure 6).

## Discussion and conclusion

The basis of this method was to use low speed centrifugation to concentrate the fat globules into a cream layer, followed by the adjustment of the underlying skim milk and then resuspension of the cream layer. In order to accurately adjust the fat content of whole milk to a specified fat amount, an equation was developed for calculating the amount of skim milk to be either removed or added. Given the relatively low RCF used for this procedure it is likely that some fat would remain in the skim and it was therefore necessary to account for the amount of fat remaining in the skim when performing this calculation.

The samples chosen for the proof of concept studies had relatively low variation of 19.3% between samples, which was reduced to 2.6% by employing the method described. For these studies, an assumed value skim milk fat content of 17 g/l was used, which was derived from the average of 72 skim milk samples centrifuged at optimal RCF. Measurement of the fat content of the skim milk would decrease the variation between samples, but was not considered to be clinically important for either the milk bank or NICU. Nonetheless, assuming a skim milk fat content did result in a large decrease in the variability of fat between samples and contributed to less than 2.2% error in the final fat content of the reconstituted milk.

The effect that centrifugation and resuspension of the milk had on the fat globule size distribution was also investigated. Low temperatures are recommended for preventing microbial growth in human milk [23], however it is not known how low temperatures affect the solidity of the cream layer and the ease of which the fat globules can be resuspended. Centrifuging at temperatures between 4°C and 10°C did not appear to affect the resuspension process. Subsequently, later centrifugation procedures were performed at 4°C to minimize any microbial growth. Fat globule size also may be important to infant gastric emptying and ability to absorb fat from the gut [24].

Centrifugation results in concentration of the fat globules into a dense cream layer at the top of the vessel, leading to the possibility of coalescence occurring and in turn altering the milk fat globule distribution. To test this hypothesis, samples of fresh and frozen milk, before and after manipulation were analyzed using laser diffraction particle sizing, a technology that has been succesfully used for studying the particle distribution of bovine milk [25]. Results were similar to that of previous findings for human milk [26,27] which employed more classical techniques such as coulter counters. Centrifugation and resuspension of milk did not alter the fat globule distribution, suggesting that coalescence does not occur under these conditions. The effect of centrifugation on milk proteins would be insignificant because much higher centrifugal forces are required for casein sedimentation [28] and the separation of small molecules (eg. lactose, oligosaccharides, peptides, hormones) from complex biological solutions cannot be achieved using centrifugation alone.

The method presented here has the potential for incorporation with current human milk banking protocols. While this study used a spectrophotometric assay for quantifying fat content of human milk, it was also demonstrated that the results from the simpler and quicker creamotocrit method correlated well with those derived from the more advanced spectrophotometric method. It is unlikely that most milk banks or NICUs would have access to a spectrophotometer, and the creamotocrit is an accurate and cost-efficient alternative for determining fat content of human milk. Using a creamotocrit it would be possible to determine milk fat content in the NICU or milk bank, standardize the fat content of the milk prior to pasteurization, followed by appropriate fortification.

The validation of this method involved using assumed values of protein and lactose. Human milk composition is challenging to quantify outside the laboratory environment. Consequently, the concentrations of nutritional components in human milk are often assumed, contributing to inaccurate nutrition of the preterm infant. In recent years, human milk analysis equipment such as the MilkoScan (FOSS International) have become available that simultaneously determines protein, lactose and fat content in human milk. The equation presented here can be expanded to include these components in relation to total energy of the milk. Ideally all the variables including fat, protein, lactose and specified energy content can be inputted into the expanded equation and in combination with current fortification regimes, a standardized fortified human milk of known energy and protein content can be prepared that precisely meets the infant's nutritional recommendations. The method is also versatile, allowing for batch processing by employing a large capacity centrifuge or alternatively, for prescriptive use for standardizing the fat content of donor milk or mother's own milk to meet the needs of a particular infant. Finally, the simplicity of this method ensures that with minimal training, non-laboratory trained staff can utilize it to standardize the energy content of breast milk for use in the NICU.

## Competing interests

The authors declare that they have no competing interests.

## Authors' contributions

CC had primary responsibility for method development and evaluation and preparation of this manuscript. PEH participated and supervised method development and evaluation and contributed to this manuscript. KS contributed to the manuscript and supervised the project.
